# Systematic Review on Microtia: Current Knowledge and Future Directions

**DOI:** 10.3390/children12040411

**Published:** 2025-03-25

**Authors:** Filippo Hellies, Silvia Fracaro, Gino Marioni, Annalisa Trotta, Martina Todesco, Martina Casarin, Andrea Bagno, Elisabetta Zanoletti, Giovanna Albertin, Laura Astolfi

**Affiliations:** 1 Bioacoustics Research Laboratory, Department of Neuroscience-DNS, University of Padova, 35128 Padova, Italy; filippo.hellies@phd.unipd.it (F.H.); silvia.fracaro@phd.unipd.it (S.F.); annalisa.trotta@studenti.univr.it (A.T.); 2 Phoniatrics and Audiology Unit, Department of Neuroscience-DNS, University of Padova, 31100 Treviso, Italy; gino.marioni@unipd.it; 3 Department of Industrial Engineering, University of Padova, Via Marzolo 9, 35131 Padova, Italy; martina.todesco.2@phd.unipd.it (M.T.); andrea.bagno@unipd.it (A.B.); 4 Department of Surgery, Oncology and Gastroenterology, University of Padova, Giustiniani 2, 35128 Padova, Italy; martina.casarin@unipd.it; 5 Section of Otorhinolaryngology-Head and Neck Surgery, Department of Neuroscience-DNS, “Azienda Ospedale Università di Padova”-University of Padova, 35128 Padova, Italy; elisabetta.zanoletti@unipd.it; 6 Section of Human Anatomy, Department of Neuroscience-DNS, University of Padova, Via Gabelli 65, 35122 Padova, Italy; giovanna.albertin@unipd.it

**Keywords:** congenital deformity, auricular reconstruction, surgical reconstruction, autologous cartilage, synthetic prostheses, 3D printing, tissue engineering

## Abstract

Background: Microtia is a congenital outer ear deformity that causes the auricle to be absent or underdeveloped. It is frequently associated with external auditory canal atresia and causes hearing and psychosocial problems. Objectives: We thoroughly investigate the aspects of microtia and explore both current and innovative therapies. Methods: A systematic literature review was conducted following PRISMA guidelines, focusing on microtia and reconstruction methodologies. This review utilized three databases: PubMed, Scopus, and Web of Science. Results: The etiology involves both genetic and environmental factors and can occur as part of a syndrome or as an isolated condition. Clinically, it has esthetic and functional implications, potentially leading to conductive hearing loss. A multidisciplinary approach is essential for treatment, which includes surgical reconstruction using autologous cartilage or synthetic prostheses. Advances in bioengineering and 3D printing offer new, less invasive solutions. Conclusions: This review aims to synthesize current knowledge on microtia, focusing on tissue engineering for auricular reconstruction, identifying research gaps, evaluating techniques, and providing a resource for clinicians to improve decision-making and foster further research.

## 1. Introduction

An underdeveloped or absent auricle is the result of a congenital malformation of the external ear known as microtia. It occurs with an incidence of approximately 1 in 6000 to 12,000 live births and can be unilateral (more common) or bilateral [1]. Microtia is estimated to affect around 1.46 percent of 10,000 children worldwide [2,3,4].

Children with this illness may suffer from psychosocial issues such as elevated anxiety, social isolation, and low self-esteem [5]. It is often associated with external auditory canal atresia, resulting in hearing problems that range from mild to severe, depending on the degree of malformation and the presence of other abnormalities in the middle and inner ear [5].

There are no definitive answers to the etiology of microtia, but it is believed to be due to a combination of genetic and environmental factors [6]. Anomalies can be isolated or associated with other syndromes, such as Goldenhar syndrome or Treacher–Collins syndrome [7]. Although studies have identified several genes that could be involved, it is challenging to establish a clear etiological framework due to the complexity of genetic and environmental interactions [8].

In clinical practice, microtia has esthetic and functional implications, with the potential for conductive hearing loss due to the absence or malformation of the auditory canal and ossicular structures [9]. Clinicians can rely on the Jahrsdoerfer grading scale as an invaluable tool in the preoperative evaluation of patients with congenital aural atresia, providing critical insights that assist in selecting the most appropriate surgical procedure [10]. The treatment of microtia requires a multidisciplinary team consisting of geneticists, otolaryngologists, audiologists, and plastic surgeons to address the functional and esthetic requirements of the patients [11]. The treatment for microtia consists of surgical interventions to reconstruct the auricle and to create or repair the auditory canal [12]. Reconstruction can be conducted with either autologous cartilage, which is typically harvested from the patient’s ribs, or with synthetic prostheses [12]. The latest developments in bioengineering and 3D printing can potentially offer more personalized and less invasive solutions for treating this condition [13].

This review of the literature is intended to synthesize current knowledge on microtia, with a particular emphasis on the use of tissue engineering for auricular reconstruction. The review is intended to identify research gaps, assess the efficacy of different tissue engineering techniques, and provide a comprehensive resource for clinicians. By highlighting recent advances and comparing traditional and innovative methods, this review hopes to assist in clinical decision-making, foster cross-disciplinary cooperation, and encourage further research and development in the field.

## 2. Materials and Methods

A systematic approach was used to identify, analyze, and synthesize the relevant literature in accordance with PRISMA guidelines. Searches were conducted in electronic databases (such as PubMed) from 10 April to 30 November 2024. To compile the reference list, MeSH terms and free-text words were used, as reported in Appendix A, filtered by past 5 years (2019–2024). The included articles were thoroughly reviewed to identify additional relevant studies. Dr. Fracaro and Dr. Hellies independently evaluated the full texts of the articles based on the inclusion criteria after removing duplicates from all titles and abstracts. Following discussions with all authors, the reviewers reached a consensus. The inclusion criteria for the studies were as follows: (i) a description of microtia in terms of pathology and epidemiology; (ii) a description of the clinical treatment of microtia and its efficacy; and (iii) an explanation of the innovative treatments proposed, such as tissue engineering. The exclusion criteria were as follows: (i) reviews and systematic reviews; (ii) non-original studies, such as recommendations, letters, editorials, conference papers, and book chapters; and (iii) studies not written in English.

## 3. Results

### Data Collection and Screening

**Figure 1:**
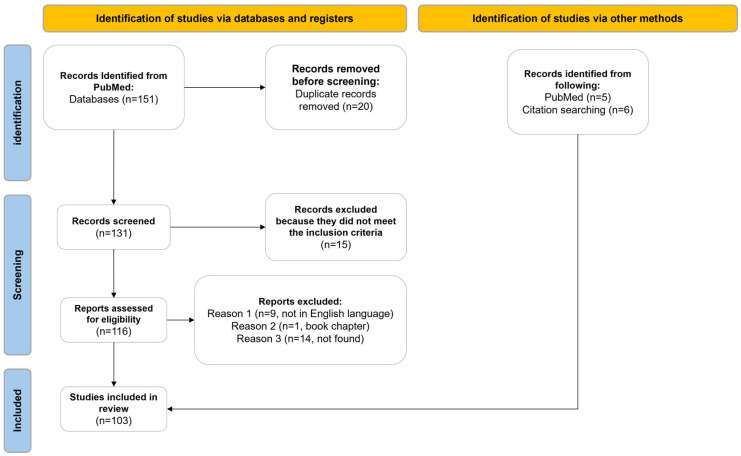
PRISMA 2020 flow diagram for this new systematic review which included searches of databases and other sources.

A total of 151 titles were retrieved from PubMed. Records not found, records not written in English, books, and duplicates were excluded from the selection process, resulting in 107 titles. By screening the full texts of these articles, we were able to apply the inclusion/exclusion criteria, excluding 15 studies and adding 11 records (articles) as potentially relevant to the topic. Ultimately, 103 records were deemed eligible for this review. The PRISMA flow diagram in Figure 1 illustrates the progression of the information through the various stages of this literature review.

## 4. Discussion

### 4.1. General Description of Microtia

Microtia is a congenital condition characterized by the abnormal development of the external ear during the fetal stage. Its severity varies from minor deformities to the complete absence of the auricle.

**Figure 2:**
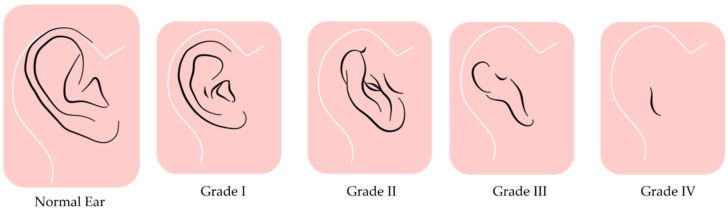
The image shows a normal ear alongside the four grades of microtia, ranging from mild structural changes (Grade I) to the complete absence of the external ear (Grade IV, anotia).

This disease is classified into four grades based on severity: Grade I involves minor malformations with nearly normal anatomy, while Grade II shows partial auricular development. Grade III presents severe hypoplasia, often with a lobular remnant, and is the most common type. Grade IV (anotia) refers to the complete absence of the external ear (Figure 2). This classification guides the development of treatment strategies [5,14,15,16].

Microtia is more prevalent in males than females, and 90% of cases are unilateral, with a predominantly diseased right side [5].

Psychosocial effects, such as depression, anxiety, and decreased self-esteem, are commonly experienced in affected patients [17].

#### 4.1.1. Development

Microtia is characterized by a malformation in the auricular cartilage, including folding, curling, or incomplete development, indicating that there are some abnormal steps in the cartilage development process.

Microtia auricular cartilage undergoes varying degrees of hypoplasia during early embryonic stages. It results from altered embryonic development and defective neural crest cell migration in the first and second branchial arches. The external ear begins to form around the sixth week of pregnancy, as six small nodules from the first and second arches merge. By the 12th week, the development of the auricle is completed [18].

Auricular cartilage exhibits distinct biochemical properties that influence its biomechanical behavior. For instance, the glycosaminoglycan content is higher in auricular cartilage, along with notable differences in the levels of hyaluronic acid and chondroitin sulfate. Additionally, auricular cartilage demonstrates a reduced elastin expression of elastin and an uneven collagen distribution, further distinguishing it from normal auricular cartilage [18].

#### 4.1.2. Genetic Studies

Approximately 40% of patients with microtia exhibit additional structural abnormalities as part of the syndrome, which may involve chromosomal abnormalities or single-gene mutations [19].

There are some genes that have been identified as directly involved in microtia, such as PLCB4, GNAI3, HOXA2, DHODH, and TBX1 [20].

HOXA2 plays a critical role in the development of the second branchial arch and mutations are linked to autosomal recessive microtia, directly affecting auricle formation.

TBX1 regulates pharyngeal arch development, which is crucial for craniofacial structures. This gene is found in 22q11.2 deletion syndrome (DiGeorge syndrome), where microtia is a common feature.

Then, there are some genes involved or indirectly involved in syndromic forms such as: ROBO1/ROBO2; SIX1/SIX5, which is linked to branchio-oto-renal (BOR) syndrome where microtia is a key feature; and CHUK, involved in embryogenesis and whose mutations can lead to developmental abnormalities, including ear defects [21].

#### 4.1.3. Epidemiological Studies

Epidemiological research has identified various factors that contribute to the development of microtia, including viral infections during early pregnancy, preeclampsia, environmental influences, and genetic predispositions.

The pathogenic mechanism of microtia is multifactorial and progresses through multiple stages. Recent genomic studies on congenital non-syndromic microtia have revealed the involvement of noncoding RNAs, such as microRNAs and long noncoding RNAs, in its development.

Increasing evidence highlights the diverse roles of ncRNAs, particularly enhancer RNAs, which are synthesized as active enhancers and influence gene expression by modulating chromatin structure and function.

Despite these findings, further research is required to validate these functions [22].

#### 4.1.4. Treatment and Tissue Engineering

Traditional auricular reconstruction relies on autologous costal cartilage, which is considered the standard due to its biocompatibility and structural stability. However, cartilage harvesting carries risks such as pneumothorax, scarring, and reduced elasticity over time.

Innovative techniques focus on bioengineered cartilage scaffolds and 3D printing, enabling the creation of customized structures with reduced invasiveness. Additionally, the use of bioengineered skin substitutes and growth factors enhances integration and improves esthetic and functional outcomes. While promising, these approaches require further studies to ensure long-term clinical efficacy [23].

### 4.2. Current Surgical Techniques in Clinical Practice, Efficacy, and Limitations

Auricular reconstruction for microtia is a complex surgical challenge that requires an integrated approach, considering technical, biological, and esthetic aspects. In recent years, several studies have introduced innovative techniques to improve functionality, esthetics, and the management of postoperative complications.

In the following section, various key aspects related to current surgical techniques in auricular reconstruction and their results in terms of efficacy and limitations will be discussed.

#### 4.2.1. Use of Autologous Cartilage

Autologous costal cartilage is considered the gold standard for auricular reconstruction in microtia due to its biological and mechanical properties, which provide a stable and long-lasting structure, ensuring a satisfactory esthetic outcome [24,25,26,27]. Its biological compatibility eliminates the risk of rejection and immunological complications, making it a reliable and widely adopted choice [28,29]. Numerous studies have confirmed its long-term efficacy, such as that of Griffin (2020), which highlights the superior mechanical properties of costal cartilage compared to other options. Griffin’s study emphasizes that costal cartilage exhibits greater stiffness and robustness, making it ideal for auricular reconstruction. In fact, costal cartilage has a significantly higher Young’s modulus of elasticity (11.43 MPa) compared to auricular cartilage (2 MPa) [24].

Recent studies, such as those by Yan (2019), Ladani (2018), and Park (2023), confirm that costal cartilage better conforms to the anatomical structure of the patient, enabling the creation of a rigid and resilient three-dimensional framework that replicates the complexity of the external ear [25,28,30]. Specifically, Yan (2019) and Ladani (2018) describe the well-established technique of harvesting cartilage from the sixth, seventh, and eighth ribs, selecting segments based on length, quality, and conformation, to construct a framework that faithfully reproduces auricular anatomy. These segments are chosen for their elasticity and strength, which allows the design of a robust support capable of adapting to the patient’s movements and ensuring long-term durability [28,30]. Beyond dimensions, the biomechanics of costal cartilage are fundamental: it must be soft enough to be sculpted yet rigid enough to maintain its shape [26,31].

Intraoperative preparation plays a crucial role in the success of the procedure. The proper dissection and management of the donor site are essential to minimize the risk of complications [28,30]. Park (2023) analyzed the long-term outcomes of secondary auricular reconstruction in patients with unsatisfactory results from primary microtia reconstruction, identifying complications such as lump deformities (91 cases), deficient convolution (19 cases), and the absence of structures with scarred mastoid skin (22 cases) [25,32]. Secondary auricular reconstruction using autologous costal cartilage yielded favorable esthetic results and manageable complications. Outcomes, evaluated using a four-point Likert scale, showed that 42% of the patients presented excellent results, while the 36% presented only good results [25].

A further significant contribution came from Asirova’s study (2023), which used CT scans to analyze the characteristics of the costal cartilage, suggesting the identification of the optimal surgical timing for reconstruction [26]. This aspect was crucial to ensure higher material quality and resistance, reducing the risk of postoperative complications and improving long-term esthetic outcomes [26]. According to this study, the optimal age for auricular reconstruction with autologous costal cartilage is from 10 years onwards, not only because the cartilage has reached an adequate size but also because the contralateral ear can be used as a model, allowing for the creation of an ear of the same size as the healthy one [26].

Autogenous cartilage also enables the detailed replication of auricular subunits through meticulous carving, reducing material requirements while achieving natural and harmonious results [33,34]. For instance, Luo et al. [29] documented successful single-stage reconstructions in 98 patients with congenital microtia, demonstrating stable and proportionate auricular morphology with minimal donor site complications. Outcomes rated as excellent or good were observed in 94 cases, with only minor issues such as hypertrophic scarring or pigmentation observed in a few patients during a 28.5-month follow-up period. Saha (2023) [35] showed a unique case of microtia associated with dextrocardia, situs inversus totalis, butterfly vertebra, and hemivertebra, describing the technical considerations necessary to optimize safety and outcomes for surgical reconstruction in such an extraordinary context. The suitability of the left or right rib cartilage for constructing the auricular frame during auricular reconstruction in pediatric patients examined by Sun (2022) [36] showed that the rib cartilage from the left side, especially the sixth rib, was generally more suitable for auricular reconstruction in pediatric patients with microtia. Anatomical abnormalities of the superficial temporal artery (STA) branches in patients with microtia and the implications for auricular reconstruction using a temporoparietal fascia (TPF) flap were described by Nguyen [37]; partial flap necrosis and facial damage were more frequent when the frontal arterial branch was within 5 mm of the facial nerve or when there was permanent nerve damage. The study of Sun (2024) [38] was a meta-analysis comparing the effectiveness of two methods of auricular reconstruction in patients with microtia: reconstruction with expanded flaps versus non-expanded flaps. The results suggested that auricular reconstruction with non-expanded flaps offers a better overall therapeutic effect, with minor postoperative complications in various key categories.

#### 4.2.2. Operative and Postoperative Complications

**Figure 3:**
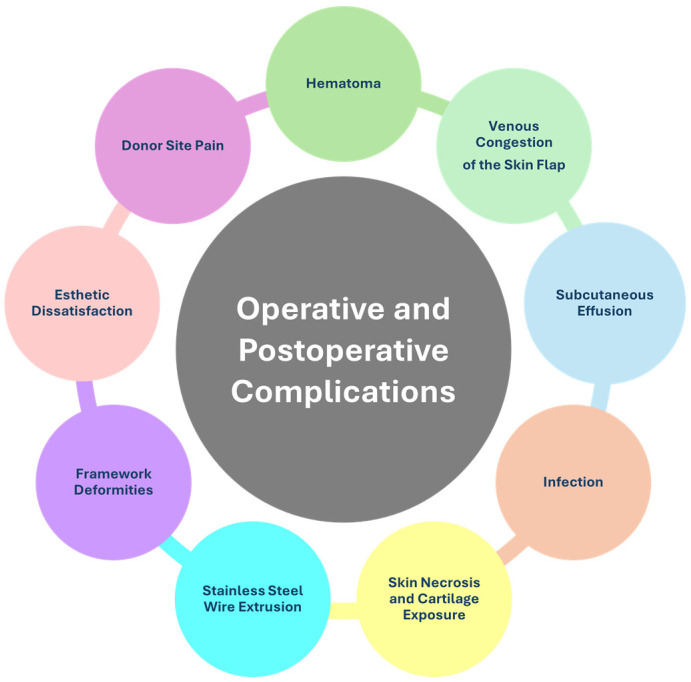
Summary of potential postoperative complications.

The use of costochondral cartilage shows positive outcomes, with a progressive reduction in complications as the surgeon gains experience. Nevertheless, complications can arise during surgery or in the postoperative period, including esthetic concerns and patient dissatisfaction (Figure 3).

The complication rate is approximately 10%, primarily occurring during the first surgical phase, consistent with other studies, such as Torres (2021), which reported a complication rate of 16% [39]. Sharma (2024) [40] analyzed early postoperative complications in patients undergoing ear reconstruction for microtia. The results showed that alloplastic implants had a higher complication rate compared to cartilage grafts (8.6% vs. 2.8%) and an increased independent risk of wound complications.

##### Hematoma

Hematoma is a common complication within the first postoperative day, caused by subcutaneous hemorrhage. To prevent this, meticulous intraoperative hemostasis, postoperative observation, and the use of suction drains are critical. The application of a compressive dressing for one day and, in some cases, hyperbaric oxygen therapy can facilitate the absorption of hematoma. This complication occurs in 1.4% of cases and accounts for 70% of all complications. Risk factors include imprecise hemostasis and unstable awakening from anesthesia. Proper surgical technique is essential [41].

##### Venous Congestion of the Skin Flap

Venous congestion manifests as a bluish discoloration occurring during the first or second postoperative day. Treatment involves small perforations with a hypodermic needle, the application of topical heparin to prevent clot formation, and hyperbaric oxygen therapy to improve circulation and recovery. This complication is attributed to the inappropriate dimensions of the subcutaneous pedicle and destruction of subcutaneous vascular networks [41].

##### Subcutaneous Effusion

A retrospective study concerning 1296 affected sides found 55 cases of subcutaneous effusion, including 24 cases using Nagata’s method and 31 cases with the expanded single-flap method. The effusion occurred within five days after drainage tube removal. The expanded single-flap method was more prone to effusion due to the greater scope of the framework compared to that of the modified Nagata method. Patients were successfully treated with the indwelling needle puncture drainage method combined with continuous negative pressure [42].

##### Infection

Infections at the surgical site typically present as abnormal erythema, swelling, abscesses, and surgical wound dehiscence, usually 1 to 2 weeks after surgery. Treatment includes drainage, irrigation, intravenous antibiotics, and hyperbaric oxygen therapy. Infections account for 0.5% of complications, with a higher risk in patients with conchal-type microtia due to insufficient sterile measures and the improper use of prophylactic antibiotics [41].

##### Skin Necrosis and Cartilage Exposure

Skin necrosis and cartilage exposure, particularly in areas such as the helix, tragus, and incisura, occur in 22.7% of cases. For exposure areas smaller than 10 mm², conservative treatments are applied, while larger areas require surgical intervention with skin flaps or grafts. This complication is associated with sharp cartilage edges, excessive protrusion, or vascular destruction during dissection [41,43,44]. Notably, these cases can occur years after surgery. For instance, one study reported a spontaneous auricular abscess with exposed cartilage 20 years post-reconstruction [45].

##### Stainless Steel Wire Extrusion

The extrusion of stainless steel wire, which is used to secure the cartilage framework during the first stage of microtia reconstruction, is another complication. This is especially common at the helix root. To reduce extrusion, thinner wires (0.20 mm instead of 0.25 mm) are recommended, with removal during follow-up or the second surgical phase. This complication represents 17% of cases and is related to the use of thick wires or improper placement [41].

##### Framework Deformities

In adult patients with calcified or fragile cartilage, fractures of the eighth costal cartilage may occur during helix construction, either intraoperatively or during follow-up. Prevention involves modifying the carving technique, using the outer edge of the framework body as the helix, and reinforcing attachment points on the upper curved section. Framework deformities represent 8.8% of complications and are caused by the improper handling of hardened cartilage, delayed follow-up, or improper patient positioning [41].

##### Esthetic Dissatisfaction

Esthetic dissatisfaction is another possible complication, often requiring secondary revision surgery. A study of 24 cases identified the importance of tailoring revision techniques to the local soft tissue conditions of the reconstructed ear to achieve satisfactory results [41,46,47].

##### Donor Site Pain and Deformities

Pain at the autologous donor site is an accessory complication. An effective solution proposed in one study involved administering local analgesics to the donor area, similarly to the techniques used for rib fractures, with good results in improving recovery time and quality of life [48]. Instead, Yang et al. [49] examined the thoracic deformities that might arise following rib cartilage harvesting for auricular reconstruction in patients with microtia. To address this issue, the authors proposed a solution involving autologous dermofat transplantation to restore chest symmetry. This technique offered an effective approach to enhance both the esthetic appearance and symmetry of the chest.

##### Excessive Hair on Reconstructed Skin

Excess hair growth on reconstructed skin is a potential esthetic issue, especially when skin expanders are used. Studies have demonstrated that both long-pulsed 800 nm diode lasers and intense pulsed light (IPL) are safe and effective methods for hair removal during all stages of ear reconstruction. Significant results have been achieved after 2–3 sessions [50,51].

#### 4.2.3. Innovative Surgical Techniques

In addition to traditional corrective techniques, several studies have explored innovative approaches for auricular reconstruction, including the use of cartilage molding, cadaveric material harvesting, and alloplastic materials such as resorbable plates or titanium supports.

##### Cartilage Molding

The application of a 3D-printed mold for the reconstruction of the auricular scaffold by using an endogenous environment was described by Wei et al. (2023) [52]. The regeneration of the constructed auricolar pavilion exploited the perichondrial flap from the sixth to eigth costal cartilage. After 6–8 months, the mold was removed, and the shaped cartilage was implanted at the final site. The results were satisfactory both esthetically and in terms of the quality of the reconstructive material. The study involved five patients, three of whom completed long-term follow-up.

##### Cadaveric Material

The harvesting of costal cartilage from cadavers represents a solution to the problem of material scarcity, particularly in children, while avoiding the need for double surgical intervention and associated complication at the donor site. A protocol was presented in which cadaveric costal cartilage was sterilized, sculpted, and subsequently implanted. The dense extracellular matrix (ECM) of allogeneic cartilage prevented lymphocyte migration into the transplanted tissue, inhibiting immune detection and limiting the immune response [53].

##### Simultaneous Reconstruction with Transcutaneous Bone Conduction Implant

Chan et al. (2019) evaluated the feasibility of simultaneous auricular reconstruction with the implantation of a bone-conduction hearing aid [54]. They showed that the approach is safe and effective and this combination might improve both esthetics and auditory function. This study presented a novel auricular prosthesis integrating a cartilage conduction hearing aid (CC-HA) using 3D data processing for patients with microtia. Preclinical evaluations showed minimal acoustic differences between the CC-HA alone and its combination with the prosthesis, with no clinical relevance. The APiCHA system offered a non-invasive, functional, and esthetic solution for hearing restoration [55].

##### Innovative Sculpting and Shaping Strategies

Advanced techniques have been proposed for the harvesting, sculpting, and utilization of costal cartilage in Grade I and Grade II microtia. Specifically, Gu et al. (2023) [56] described a method for the reconstruction of the helix and antihelix, which ensured the good preservation of the costal perichondrium and the use of resorbable sutures. The esthetic outcome was judged satisfactory by 100% of the 65 patients, without significant complications.

Other researchers proposed personalized modifications applied to the sculpting of different ear parts, based on the individual patients’ characteristics [57]. These modifications upgraded structural stability, esthetic appearance, and the durability of the results during follow-up. Some complications had to be managed postoperatively. The described techniques offered flexibility and adaptability but required further measures to minimize postoperative risks and ensure symmetry in the final result. An alternative method was reported by Park et al. (2021) [58] using a V-Y advancement of a temporal triangular flap to obtain additional soft tissue without the use of a tissue expander. There were no specific complications after the first surgical stage, but one patient had complications after the second stage (auricular elevation). The authors reported that most of the patients reported excellent esthetic results, with satisfactory convolution. The average number of operations required to complete the reconstruction was two, lower than the conventional method using a tissue expander. Other researchers evaluated the clinical efficacy of a surgical method combining a cross-flap with autologous auricular cartilage grafting in the treatment of type I-III microtia [59]. The proposed method was effective in treating type I-III microtia, making this surgical approach feasible for correcting this deformity. In the study [60], the issue of “lop ear” was addressed, characterized by an insufficient helix and scapha, a poorly developed antihelix, and helix folding. Methods to resolve this issue were proposed based on the severity of “lop ear”. More severe cases of “lop ear” were considered a type of conchal microtia. Lesta Compagnucci et al. described separating the implanted cartilaginous framework using fascial flaps and a dermal template to create an auriculocephalic sulcus. This technique reduced operative time, preserved fascia for future use, and minimized scarring with similar complication rates [61].

##### Plates and Titanium Supports

Li et al. (2023) proposed an innovative technique to improve ear projection and elevation in microtia reconstruction, utilizing a retroauricular fascial flap that enveloped two titanium structures [62]. These structures served as supports, aiding in achieving a stable and well-defined projection of the reconstructed ear. In this way, exclusive reliance on costal cartilage, which may not always be sufficient, was avoided. The technique, performed in a total of 51 patients with unilateral microtia (age range: 9–35 years), demonstrated satisfactory results in creating an esthetically pleasing ear with solid posterior elevation and natural projection [62].

##### Material Quantification

Li et al. (2024) proposed a method for quantifying the available costal cartilage in patients to optimize the surgical process and reduce the risk of material insufficiency during harvesting [63].

#### 4.2.4. Surgical Advances for Esthetic Improvement

In recent years, surgical techniques for ear reconstruction in patients with microtia have made significant advances, aimed at improving esthetic and functional outcomes, reducing complications, and optimizing long-term stability. The most notable innovation was the introduction of the posterior auricular artery perforator (PAAP) free flap, described by Banda (2020), which enhanced the vascularization of the reconstructed tissues, reducing the risk of necrosis and improving the stability of the auricular implant [64]. This technique was particularly effective in the reconstruction of the retroauricular sulcus, a critical area for achieving functional and esthetically pleasing results [64].

Simultaneously, innovations in cartilage framework sculpting techniques have led to significant esthetic improvements. Luo et al. (2023), in a study concerning 98 patients, introduced modifications to the sculpting of costal cartilage, optimizing the definition of the helix and antihelix, as well as the angulation and symmetry of the auricular pavilion [29]. These improvements allowed for reconstructions more faithful to the natural anatomy of the ear, positively affecting both the esthetic appearance and functionality of the pavilion [29]. Similarly, Mazeed et al. (2020) proposed the use of a complete ring-shaped cartilage framework with accentuated curves, which improved structural stability and definition of the tragus and concha, preventing postoperative deformities and optimizing esthetic results [65].

The use of costal cartilage continues to be a fundamental resource in ear reconstruction. Wang et al. (2021) introduced a crescent-shaped cartilage graft to improve the retroauricular contour [66], or a three-stage technique involving the creation of a cartilage framework and the use of a skin flap, demonstrating satisfactory esthetic and functional results despite the inherent challenges in adult ear reconstruction [67].

An innovation of Firmin’s technique improved earring insertion in patients undergoing ear reconstruction [68]. This modification allowed for the creation of a specific area for the earring hole without compromising esthetics, thereby improving patient satisfaction and postoperative quality of life [68].

Other innovative approaches include the study by Lee et al. (2023), which integrated a superficial temporal fascial flap with a costal cartilage graft, improving the vascularization and stability of the auricular framework [69]; a three-stage approach showed superior results in the study by Xu et al. (2019), who developed advanced three-dimensional modeling of the cartilage framework, leading to significant improvements in esthetic results and a reduction in complications [70]. Furthermore, studies conducted by Ibrahiem (2023) optimized conchal-type microtia reconstruction and the elevation of the ear [71] with improvements in esthetics and functionality and a reduction in scarring [72,73]. Greene et al. [74] proposed an alternative approach for ear elevation, where an advanced x-y flap improved the integrity and contour of the reconstructed auricular sulcus and a modification to the Nagata technique improved the projection and esthetics of reconstructed ears [75]. This study involved 38 patients (39 ears), whose ages ranged from 6 to 34 years, with an average follow-up of approximately 33.6 months. A proportion of 15.4% of the results were rated as excellent and 41% as good. A total of 79.9% of patients or their families reported being satisfied. No cases of flap necrosis or hypertrophic scars were observed. Low complication rates were found: a mismatch in skin color (7.7%), unacceptable thickness (5.1%), and the presence of hair or stretch marks (10.3%). Other researchers focused on auricular reconstruction, particularly optimizing rib cartilage carving techniques to create esthetically harmonious auricular structures. In 406 patients, 112 ears were rated as “excellent”. A design based on these data allowed for the creation of a harmonious and well-defined three-dimensional ear, with minimal risk of complications [76].

#### 4.2.5. Tissue Expansion

In recent years, the combination of tissue expansion and advanced reconstructive techniques has significantly improved the management of microtia by optimizing the use of available tissues and reducing the need for skin grafts. These techniques have also improved the esthetic and functional outcomes of auricular reconstruction.

An innovative approach developed by Wang et al. (2021) combined tissue expansion with autologous auricular reconstruction. The technique, applied to 104 patients (average age of 12 years), involved two stages: the use of an expander to stretch the mastoid skin, followed by a Y-shaped incision to separate the earlobe from other skin structures. In the second stage, a cartilage framework was inserted into the expanded area, optimizing the projection and definition of the cranio-auricular sulcus. This approach reduced complications and improved patient satisfaction [77].

A similar technique developed by Chen et al. (2020) employed an extended postauricular flap combined with tissue expansion, eliminating the need for skin grafts and reducing visible scarring. Applied to 106 patients, this technique achieved a success rate of 93.4%, improving vascularization and reducing complications compared to traditional methods [78].

Liang et al. (2023) optimized the design and positioning of tissue expanders, improving skin coverage and esthetic outcomes, as this technique reduced skin necrosis and enhanced tissue integration, resulting in a high degree of patient satisfaction [79].

The tissue expansion technique for auricular reconstruction has several limitations, including a prolonged treatment period, high saline volume requirements, and restricted patient selection based on skin elasticity and thickness. Reduced vascularization of the expanded flap increases the risk of necrosis and ischemia, while excessive tissue expansion may promote hair growth in the reconstructed area. Additionally, insufficient cartilage rigidity can compromise the definition of the auriculocephalic sulcus. The risk of infections, the need for corrective procedures, and the multiple surgical stages make this technique complex and less widely applicable [77,78,79].

### 4.3. Tissue Engineering in Otology Application

A tissue engineering approach to auricular reconstructions could offer the potential to overcome the limitations of both autologous and alloplastic transplants.

Tissue engineering has facilitated the creation of cartilaginous frameworks tailored to individual patient needs. However, several challenges persist, including the requirement for large quantities of chondrogenic cells, addressing the complexities of resistance to deformation forces, and ensuring long-term framework stability [1]. It is important to note that auricular cartilage is predominantly composed of collagen and elastin fibers embedded in a proteoglycan gel, imparting viscoelastic properties to the structure [1]. For this reason, the graft must not only meet structural demands concerning the shape substitute, including its dimensions and thickness, but also replicate the mechanical properties of the auricle, which has been demonstrated to have different stiffnesses across different regions [24].

**Figure 4:**
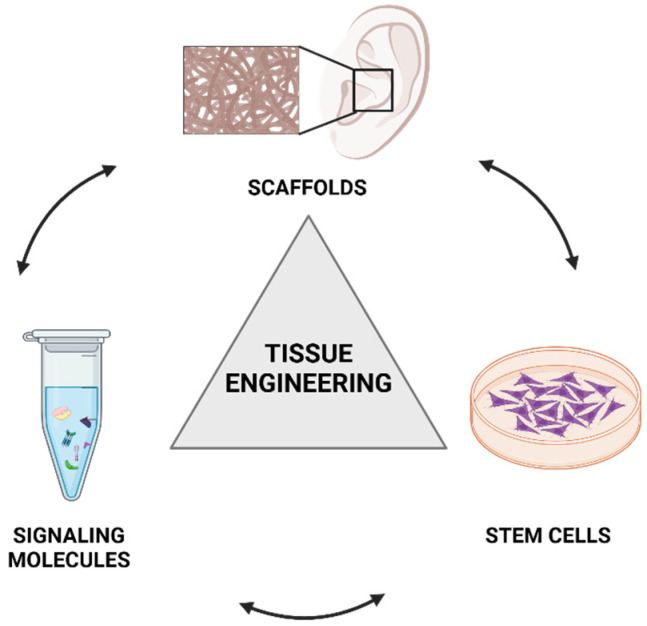
The tissue engineering triad: scaffolds, cells, and signaling molecules are the three key elements of tissue engineering. Scaffolds act as the structural framework for tissue formation, cells play a role in tissue regeneration, and signaling molecules regulate cellular activities and interactions. Created in BioRender. https://BioRender.com/i36c115, accessed on 28 February 2025.

Thus, to closely mimic native tissue, an ideal scaffold should possess several key characteristics, such as an adequate pore size, a large surface area, a degradation rate balanced with the rate of tissue regeneration, sufficient mechanical strength to maintain its predesigned shape, biocompatibility, and positive interactions with cells. These include promoting cellular adhesion, migration, proliferation, and differentiation to achieve optimal integration between the biomaterial and tissue (Figure 4).

For this reason, one of the most promising approaches involves directly fabricating the 3D shape [80] of the ear using the scaffold material, potentially incorporating cells within the construct. While these strategies show promise, they are not without limitations. However, the major challenge in engineered cartilage for auricular reconstruction is the exceptionally high number of chondrocytes required. Cartilage from microtia patients does not provide a sufficient number of functional chondrocytes to meet this demand. Furthermore, even when chondrocytes are cultured in monolayers, achieving the necessary amount remains problematic due to the progressive loss of their chondrogenic potential during the in vitro expansion [1].

Regarding 3D reconstruction, a current procedure involves the reuse of microtic tissues. Specifically, vestigial cartilage can successfully replace costal cartilage to enhance the projection and three-dimensional contour of the reconstructed ear, yielding satisfactory results in terms of shape, symmetry, and stability, with high patient satisfaction [81]. Additionally, Jacob et al. found that microtic perichondrocytes exhibit similar chondrogenic properties to chondrocytes in vitro, suggesting their potential as a promising cell source for auricular reconstruction [6]. However, results are contradictory; while some studies report robust cartilage formation by microtic chondrocytes, others demonstrate their inferior chondrogenic capacity and the formation of disorganized structures in 3D cell culture models [17].

**Figure 5:**
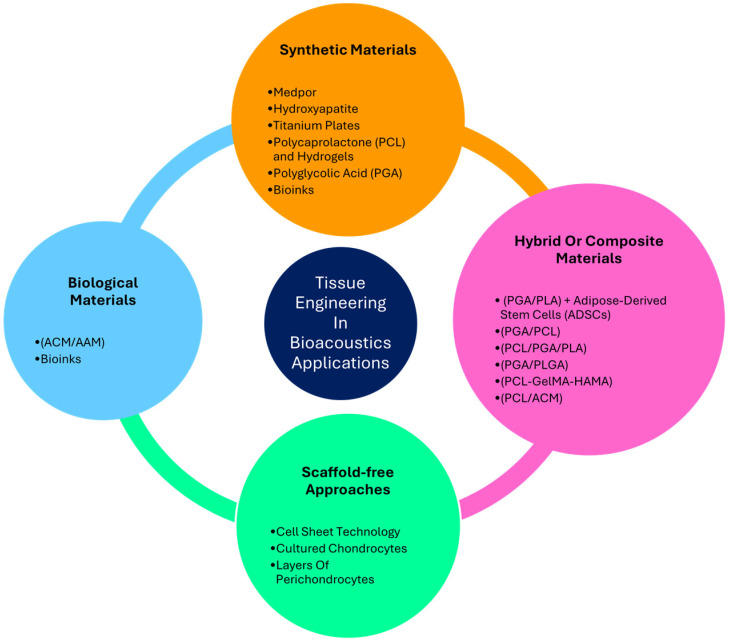
Overview of materials used in bioacoustics, including synthetic biomaterials, hybrid and composite materials, and scaffold-free approach for tissue engineering applications.

In the following sections, synthetic, hybrid/composite, and biological materials utilized for external ear reconstruction will be described, along with their respective advantages and limitations. Additionally, scaffold-free techniques, which represent an alternative approach in tissue-engineered auricular reconstruction, will also be discussed (Figure 5).

#### 4.3.1. Synthetic Materials

Synthetic materials have emerged as alternatives to autologous rib cartilage for auricular reconstruction, addressing the issue of donor site morbidity associated with cartilage harvesting. Among these, porous high-density polyethylene (pHDPE), commercially known as Medpor (Stryker, Kalamazoo, MI, USA), is one of the most extensively utilized materials. Its interconnected pore structure (average pore size ~150 µm) promotes fibrovascular ingrowth, facilitating integration with surrounding tissues. Compared to autologous cartilage, pHDPE offers advantages such as reduced operative time, the possibility of use in younger patients, and the elimination of complications related to rib cartilage harvesting [82]. A 3D mirror-image prefabricated template served as a three-dimensional reference for surgeons to meticulously sculpt the MEDPOR framework. This step ensured that the final implant closely matched the intricate details and spatial relationships of the patient’s normal ear, enhancing both precision and symmetry in the reconstruction process [83]. However, compared to autologous cartilage, Medpor exhibits lower biocompatibility, as it lacks the ability to integrate dynamically with the surrounding biological environment. Unlike native tissue, it does not undergo remodeling or adapt to physiological changes over time. Additionally, Medpor does not grow with the patient, which may limit its suitability for pediatric applications, where long-term tissue adaptation is crucial for optimal functional and esthetic outcomes.

Wei et al. (2023) [84] proposed a novel method for repairing cartilaginous defects by using hyaluronic acid (HA) and leveraging the self-regenerative potential of the intercostal rib space. HA was used to 3D print auricular models, which were then implanted in the chest wall to promote tissue repopulation. An incision was made between the sixth and eighth ribs, and the perichondrium was detached from the costal cartilage. The costal cartilage from the 6th to 8th ribs was removed, and the perichondria were sutured together to form a connected perichondral flap. The HA ear-shaped mold was then sutured to this flap. After 6–8 months, the HA molds were removed. The histological examination of the explanted tissue revealed ear-shaped, cartilage-like structures, with H&E staining showing the presence of chondrocytes. Alcian blue staining indicated a rich matrix of glycosaminoglycans (GAGs). Immunohistochemistry with anti-collagen type II antibodies demonstrated that GAG-positive regions also contained collagen type II. The study reported no significant adverse events from implanting the HA mold into the chest cavity, and no adverse reactions were observed even when the auricular implant was used as a replacement for a damaged ear [84].

An important factor when selecting a support material is its suitability for pediatric patients, who constitute a substantial proportion of recipients. Unlike HA or titanium plates, resorbable plates do not require removal, which represents a critical advantage.

While the mechanical strength of resorbable plates is lower compared to HA or titanium, this limitation is less consequential in auricular reconstruction due to the minimal physical stress exerted on the plates [84].

Resorbable plates are recognized for their favorable attributes, offering robust and long-lasting support without excessive volume. They provide reliable fixation with a reduced risk of dislocation when secured with screws. To avoid the drawbacks of previous synthetic materials, biodegradable scaffolds, such as those combining polymers like polycaprolactone (PCL) with hydrogels, were explored to promote tissue regeneration while providing temporary mechanical support. These materials degraded over time as new cartilage formed, avoiding the long-term presence of foreign materials [85].

In detail, the PCL was used for ear reconstruction by using a 3D printing technique mimicking the real cartilage, where pores of 2 mm were incorporated to enable the placement of diced cartilage, which facilitated its regeneration. The same technique was applied in a clinical trial with five patients with unilateral microtia who underwent a two-stage ear reconstruction [85]. The 3D-printed cartilage, designed to be non-identical to the other ear graft, demonstrated to provide appropriate vascularity and a reduced infection risk [85].

Another biodegradable material is polyglycolic acid (PGA), which has a fibrous structure with a large surface area and significant pore volume, facilitating tissue integration and cell regeneration, particularly chondrocyte growth. The degradation byproduct, glycolic acid, is non-toxic and is metabolized through the tricarboxylic acid cycle, ultimately being excreted as water and carbon dioxide, with a portion also eliminated in the urine. For this reason, PGA was selected as the suitable scaffold for chondrocyte culture, subcutaneous implantation, and cartilage development, aiming to optimize the number of passages and environmental conditions to promote the growth of auricular cartilage from a folded PGA scaffold while preserving its biofunctional properties [86]. In the study, the optimization of autologous chondrocyte seeded PGA scaffolds was investigated for ear cartilage regeneration starting from a small ear biopsy. In particular, PGA scaffolds seeded with chondrocyte + mesenchymal stem cells (MSCs) subcutaneously implanted in immunosuppressed mice did not grow after two months of implantation, but when the same material seeded with chondrocytes was implanted in rabbits, cartilage 10 times larger than the original PGA scaffold formed, with biofunctional and mechanical properties similar to ear cartilage. For ear reconstruction, an innovative approach based on 3D printing was recently proposed, enabling the creation of patient-specific auricular structures to closely mimic the unique anatomy of the individual. Imaging techniques such as CT or MRI scans of the healthy contralateral ear can generate highly personalized 3D models for reconstruction purposes. These methods allow for the more precise replication of natural ear morphology, enhancing esthetic and functional outcomes. One of the primary challenges in 3D bioprinting for auricular reconstruction lies in balancing the scaffold’s structural integrity with its ability to support cell migration and tissue integration. Materials like bioinks composed, for example, of hydrogels are commonly used due to their biocompatibility and capacity to encapsulate cells like chondrocytes that are responsible for cartilage production [85].

The framework for 3D-printed ears has been demonstrated to offer several advantages compared to autologous costal cartilage or synthetic alternatives like porous polyethylene. First, it allows for the precise, patient-specific modeling of the ear, facilitated by CT scans that generate highly accurate designs. This method enables the detailed extraction of unaffected ear structures by differentiating surrounding tissues and isolating specific auricular cartilage regions [85]. Second, there is no risk of complications related to donor sites. Third, the choice of implant materials can be tailored to achieve the desired balance between mechanical strength and biodegradability. Additionally, the approach ensures the preservation of the subcutaneous pedicle, enhances the vascularity of the skin flap, and lowers the likelihood of postoperative issues such as infections, skin necrosis, or implant exposure. Lastly, PCL-based 3D printing for auricular the reconstruction of the cartilage delivers robust mechanical properties with gradual biodegradability, making it a highly effective solution [86].

Poly-ε-caprolactone was used by Velasquillo et al. (2024) [87] as well. In detail, 3D-printed Poly-ε-caprolactone scaffolds coated with a fibrin hydrogel and seeded with human microtia chondrocytes were subcutaneously implanted into the dorsal region of 10 athymic mice. As a control group, five mice received scaffolds coated only with fibrin hydrogel, without chondrocytes. Four weeks after implantation, the authors reported no signs of extrusion or skin necrosis, and the ear-like structures remained stable at the implantation site [87].

Specifically, in the seeded group, white cartilage-like tissue was formed, exhibiting sufficient mechanical strength to retain its initial shape despite the pressure exerted by the overlying skin. Additionally, the grafts in this group were covered with a thin layer of vascularized tissue that resembled native tissue. Conversely, in the unseeded group, no new tissue formation was observed, and the fibrin hydrogel had resorbed. However, the grafts showed no signs of degradation but were notably more rigid compared to those of the seeded group [87].

#### 4.3.2. Hybrid or Composite Materials

Hybrid materials represent a specific subset within composite materials. Composites, by definition, are created by combining two or more distinct materials that differ in their chemical and/or physical properties, resulting in a new material with enhanced characteristics tailored for a specific application. A widely accepted description of hybrid materials highlights the synergistic integration of organic and inorganic components at the microscopic (molecular) level, leading to the development of unique material properties. The close integration of inorganic, organic, or a combination of both types of components enables the formation of advanced materials with improved functionalities compared to the properties of their individual constituents [88].

In auricle reconstruction, polyglycolic acid/polylactic acid (PGA/PLA) composite scaffolds have gained significant attention in tissue-engineered ear reconstruction due to their versatility and ease of molding. For example, in a canine model, PGA demonstrated promising results in terms of cell adhesion compared to other biodegradable polymers, promoting cartilage regeneration. However, it did not exhibit entirely satisfactory mechanical properties, as it failed to maintain the complex three-dimensional shape of a normal ear. For this reason, the combination of PGA with PCL was investigated to merge the positive attributes of both materials [89], creating a hybrid scaffold as stated elsewhere [88]. Specifically, a PCL scaffold with suitable rigidity and ear-like geometry was coated with PGA nanofibers. Nanofibers were chosen over conventional PGA because it was shown that PGA, during hydrolysis, increased environmental acidity, leading to inflammation and localized tissue destruction impeding regeneration. In contrast, the PGA/PCL material treated with ethanol improved hydrophilicity and thus cell adhesion to the scaffold. Thus, PGA/PCL might have facilitated cartilage regeneration, while untreated scaffolds led to a progressive loss of chondrocytes and proteoglycans [89].

Wu et al. (2024) [90] further advanced this technique by repopulating PGA/PLA scaffolds with adipose-derived stem cells (ADSCs), which are readily accessible, abundant, and possess strong chondrogenic potential. These ADSCs, co-cultured with chondrocytes overexpressing RhoA, demonstrated enhanced extracellular matrix (ECM) deposition and improved chondrocyte organization. The overexpression of RhoA in microtia chondrocytes significantly promoted cell migration, facilitating more uniform tissue formation. Coculturing mesenchymal stem cells and chondrocytes has consistently been shown to be a superior strategy compared to using individual cell types, as it leverages the synergistic effects of both cell populations in cartilage tissue engineering. In their study, Wu et al. implanted PGA/PLA constructs populated with modified ADSCs and MSCs subcutaneously in nude mice. After four weeks of preculture in vitro, the scaffolds exhibited the significant degradation of PGA/PLA fibers, leaving behind well-organized tubular structures and cartilage-like tissue. Histological analysis revealed a highly organized chondrocyte arrangement in constructs utilizing RhoA-overexpressed cells, further demonstrating the potential of this approach for auricular cartilage regeneration.

Resorbable plates were then used in a two-stage microtia reconstruction for 12 patients to provide structural support during auricular elevation [91]. The plates were gradually resorbed, eliminating the need for rigid materials such as metals or autologous cartilage. This approach resulted in a well-defined contour and appropriate elevation of the ear, with promising long-term outcomes, including a low complication rate and high patient satisfaction. In another study, Mukherjee et al. (2021) [8] aimed to improve the biocompatibility of PLA by developing scaffolds composed of polycaprolactone (PCL), gelatin methacrylate (GelMA), and hyaluronic acid methacrylate (HAMA) to address the limitations of traditional PCL-based materials. These scaffolds were enzymatically treated with lipase to enhance porosity, promote accelerated in vivo degradation, and improve biocompatibility compared to that of untreated PCL, used as a control. The materials were evaluated in vivo by subdermal implantation in sheep, representing a non-functional test. While there was no skin irritation, the formation of a pseudocapsule and limited tissue integration were observed, alongside a modest inflammatory response characterized by multinucleated giant cells surrounding the scaffolds. Importantly, the PCL-GelMA-HAMA scaffolds demonstrated greater cell repopulation compared to pure PCL. Despite this improvement, both materials elicited an inflammatory response, highlighting the need for further optimization to reduce adverse reactions while maintaining biocompatibility and promoting effective tissue integration [8].

Moreover, hybrid materials have recently gained attention as a promising approach to address both mechanical and biological challenges using the 3D printing approach. This technique combines the precision and strength of a stiffer polymer for structural support with biologically derived materials that create a favorable microenvironment for cell proliferation and differentiation, including stem cells. By co-printing these components, hybrid printing ensures both mechanical integrity and the biological functionality necessary for effective tissue regeneration. Despite these successes, the presence of heterogeneous cartilage regions and residual fibrous tissue highlights the need for further optimization of scaffold preparation techniques and a comprehensive evaluation of their biosafety. These findings underscore the importance of fine-tuning bioink formulations and enhancing printing methodologies to achieve uniform cartilage regeneration and minimize adverse tissue responses [88].

#### 4.3.3. Biological Materials

Auricular cartilage consists of an extracellular matrix (ECM) rich in type II collagen, proteoglycans, elastin, and water, providing structural strength and elasticity. Type II collagen forms the primary structural framework, while elastin fibers, arranged in a fine network interspersed with collagen fibrils, contribute to the deformability essential for auricular function. Mimicking this composition is a key objective in the development of biological substitutes for auricular cartilage reconstruction. By replicating these properties, it is possible to create a replacement that closely resembles native cartilage, providing an effective solution for restoring cartilage in conditions such as microtia.

To study the state of cartilage, various techniques can be employed, as demonstrated in the study of elastic and hyaline cartilage derived from laryngeal cartilage. In Gentilin et al. (2023), a protocol for whole-organ decellularization was developed to preserve the integrity of the ECM [92]. The efficiency of decellularization and the preservation of ECM components were evaluated using a range of staining and microscopy techniques. Hematoxylin/eosin (H&E), Masson Trichrome, and DAPI staining, along with DNA quantification, were utilized to assess decellularization efficiency. For collagen detection and quantitative assessment, Van Gieson staining, Picrosirius Red staining (PRS) and multiphoton laser scanning microscopy (MPM) were employed. Additionally, polarized PRS was used to investigate the collagen network, while Weigert staining and MPM were applied for elastin detection and quantification. This comprehensive approach ensured a thorough evaluation of the decellularization process and the preservation of critical ECM components [92].

Collagen, the most prevalent component of the ECM, has emerged as a promising biomaterial for scaffolds in cartilage reconstruction, particularly in cases such as auricular repair for microtia. Its advantages include low immunogenicity, a porous structure conducive to cell infiltration, and excellent biocompatibility. However, collagen-based scaffolds often lack the mechanical stiffness required for the structural demands of auricular cartilage. Additionally, biologically derived tissues, such as the decellularized cartilage matrix (ACM) and allograft adipose matrix (AAM), represent some of the most advanced materials under investigation for auricular tissue engineering. In recent years, decellularized tissues have garnered significant interest due to their promising applications in various fields of tissue reconstruction. The decellularization process involves the removal of cellular components from xenogeneic tissues, which are readily available in large quantities. This approach renders the material non-immunogenic while preserving the extracellular matrix structure, making it suitable for recellularization with the patient’s own cells. Such properties make decellularized tissues particularly attractive for the development of scaffolds that combine biocompatibility, mechanical stability, and the potential for functional integration [93,94,95,96,97,98].

In detail, for the reconstruction of ears affected by microtia, decellularized adipose tissue was tested in the form of disks measuring 15 mm in diameter and 2 to 3 mm in thickness as a scaffold for the co-seeding of porcine auricular chondrocytes and stem cells derived from cadavers’ adipose tissue (two ratios were evaluated, specifically 1:5 and 1:9) [99,100,101,102]. Compared to the two control groups (i.e., each cell type seeded individually on the scaffold), which remained viable even 10 weeks post-seeding, the co-seeding of chondrocytes and adipose tissue-derived stem cells facilitated the deposition of the ECM, particularly collagen type II. Among the tested ratios, the 1:9 ratio (chondrocytes–adipose tissue-derived stem cells) yielded the most favorable results [96].

#### 4.3.4. Scaffold-Free Approach

Scaffold-free techniques have been introduced for cartilage engineering, including cell sheet technology, aggregate culture methods, and self-assembly processes. Aggregate culturing involves cultivating cells in a way that facilitates cell-to-cell interactions, resulting in the formation of free-floating cell clusters in a two-dimensional configuration. Cell sheet technology, instead, employs cultured monolayers that are manipulated thermally or physically, sometimes with or without enzymatic cleavage, depending on the mandrel used. This method enables the stacking of multiple layers resulting in a thicker tissue construct. In contrast, the self-assembly process enables chondrocytes to aggregate naturally without external energy input. This approach leverages intrinsic free energy and cellular tension to promote adhesion between cells, simulating the mesenchymal condensation observed during natural cartilage development [94,96,99]. It should be noted that the scaffold-free approach generates a cartilage block that requires surgical sculpting to achieve the desired shape, rather than a preformed three-dimensional auricular structure. This method produces cartilage without the use of external supporting materials; however, the resulting tissue necessitates additional shaping to attain the final anatomical configuration.

Cultured chondrocytes obtained from autologous cartilage represent a potential alternative to the traditional use of rib cartilage in auricular reconstruction. Protocols have been developed for extracting chondrocytes from small tissue samples, expanding them in vitro, and generating cartilage constructs suitable for implantation. Despite these advancements, studies using immunosuppressed mice to test chondrocyte-laden scaffolds or hydrogels (e.g., PGA, polylactic acid, or their blends) have not successfully produced the cartilage volume required to recreate a full-sized human ear. This shortfall is partly attributed to the limited regenerative capacity of chondrocytes from microtia tissue compared to those from healthy cartilage. To overcome these challenges, it is essential to refine cell expansion methods, optimize passage numbers, and establish ideal culture conditions for the effective use of microtia-derived autologous chondrocytes in tissue-engineered scaffolds [99].

Enomura et al. (2020) [103] proposed the use of three layers of perichondrocytes harvested from microtia patients to create a scaffold-free construct capable of regenerating elastic cartilage when implanted in NOD/SCID mice. Although promising, this approach resulted in the formation of cartilage measuring only 1 cm^3^, whereas a cartilage volume of 10 cm^3^ is required to effectively treat microtia [100].

## 5. Conclusions

Microtia reconstruction remains a challenging yet evolving field that bridges traditional surgical techniques with groundbreaking advancements in tissue engineering and regenerative medicine. While autologous costal cartilage grafting has long been regarded as the gold standard due to its biocompatibility and structural stability, its limitations—such as donor-site morbidity, technical complexity, and variability in outcomes—highlight the critical need for innovative alternatives.

Recent advancements in tissue engineering, including 3D bioprinting, scaffold-based constructs, and stem cell therapies, are transforming the landscape of auricular reconstruction. These technologies offer precise, personalized solutions that reduce invasiveness, improve esthetic outcomes, and minimize complications. For instance, bioengineered scaffolds and decellularized matrices provide platforms for cartilage regeneration, while 3D-printed models enable preoperative planning and mirror-image reconstructions that enhance symmetry and patient satisfaction. Additionally, the integration of endogenous stem cells and growth factors holds immense potential for promoting tissue regeneration and long-term stability.

Despite these promising developments, significant challenges remain. The standardization of protocols, optimization of biomaterials, and validation of long-term clinical outcomes are essential to ensure the safety and efficacy of these emerging techniques. Furthermore, broader accessibility to advanced technologies, particularly in regions with high microtia incidence, must be addressed to maximize their global impact.

In summary, this review underscores the transformative potential of tissue engineering in microtia reconstruction, emphasizing its role in overcoming the limitations of traditional methods. By advancing regenerative approaches and integrating cutting-edge technologies, we can pave the way for more effective, less invasive, and highly personalized treatments, ultimately improving quality of life for patients with microtia.

## Appendix A

Specific search details were entered into the query boxes of each database as follows.

Pub med

(((ear cartilage) AND (congenital microtia)) OR ((“Congenital Microtia”[Mesh]) AND (“Ear Cartilage”[Mesh]))) OR (((“Congenital Microtia”[Mesh]) AND (“Tissue Engineering”[Mesh])) OR ((congenital microtia) AND (tissue engineering))) AND ((y_5[Filter]) AND (english[Filter])) AND ((y_5[Filter]) AND (english[Filter]))

Scopus

(TITLE-ABS-KEY (congenital AND microtia) AND TITLE-ABS-KEY (ear AND cartilage)) OR (TITLE-ABS-KEY (congenital AND microtia) AND TITLE-ABS-KEY (tissue AND engineering)) AND PUBYEAR > 2018 AND PUBYEAR < 2025 AND (LIMIT-TO (DOCTYPE, “ar”)) AND (LIMIT-TO (LANGUAGE, “English”))

Web of Science

Congenital microtia (All Fields) and ear cartilage (All Fields) or congenital microtia (All Fields) and tissue engineering (Author) and 2020 or 2021 or 2022 or 2023 or 2024 or 2019 (Publication Years) and Article (Document Types) and English (Languages)
